# Estimating Phred scores of Illumina base calls by logistic regression and sparse modeling

**DOI:** 10.1186/s12859-017-1743-4

**Published:** 2017-07-11

**Authors:** Sheng Zhang, Bo Wang, Lin Wan, Lei M. Li

**Affiliations:** 1 National Center of Mathematics and Interdisciplinary Sciences, Academy of Mathematics and Systems Science, Chinese Academy of Sciences, Beijing, 100190 China; 2 University of Chinese Academy of Sciences, Beijing, 100049 China

**Keywords:** Base-calling, Logistic regression, Quality score, *L*_1_ regularization, AIC, BIC, Empirical discrimination power

## Abstract

**Background:**

Phred quality scores are essential for downstream DNA analysis such as SNP detection and DNA assembly. Thus a valid model to define them is indispensable for any base-calling software. Recently, we developed the base-caller 3Dec for Illumina sequencing platforms, which reduces base-calling errors by 44-69% compared to the existing ones. However, the model to predict its quality scores has not been fully investigated yet.

**Results:**

In this study, we used logistic regression models to evaluate quality scores from predictive features, which include different aspects of the sequencing signals as well as local DNA contents. Sparse models were further obtained by three methods: the backward deletion with either AIC or BIC and the *L*_1_ regularization learning method. The *L*_1_-regularized one was then compared with the Illumina scoring method.

**Conclusions:**

The *L*_1_-regularized logistic regression improves the empirical discrimination power by as large as 14 and 25% respectively for two kinds of preprocessed sequencing signals, compared to the Illumina scoring method. Namely, the *L*_1_ method identifies more base calls of high fidelity. Computationally, the *L*_1_ method can handle large dataset and is efficient enough for daily sequencing. Meanwhile, the logistic model resulted from BIC is more interpretable. The modeling suggested that the most prominent quenching pattern in the current chemistry of Illumina occurred at the dinucleotide “GT”. Besides, nucleotides were more likely to be miscalled as the previous bases if the preceding ones were not “G”. It suggested that the phasing effect of bases after “G” was somewhat different from those after other nucleotide types.

**Electronic supplementary material:**

The online version of this article (doi:10.1186/s12859-017-1743-4) contains supplementary material, which is available to authorized users.

## Background

High-throughput sequencing technology identifies the nucleotide sequences of millions of DNA molecules simultaneously [1]. Its advent in the last decade greatly accelerated biological and medical research and has led to many exciting scientific discoveries. Base calling is the data processing part that reconstructs target DNA sequences from fluorescence intensities or electric signals generated by sequencing machines. Since the influential work of Phred scores [2] in the Sanger sequencing era, it has become an industry standard that base calling software output an error probability, in the form of a quality score, for each base call. The probabilistic interpretation of quality scores allows fair integration of different sequencing reads, possibly from different runs or even from different labs, in the downstream DNA analysis such as SNP detection and DNA assembly [3]. Thus a valid model to define Phred scores is indispensable for any base-calling software.

Many existing base-calling software for high throughput sequencing define quality scores according to the Phred framework [2], which transforms the values of several predictive features of sequencing traces to a probability based on a lookup table. Such a lookup table is obtained by training on data sets of sufficiently large sizes. To keep the size of lookup table in control, the number of predictive features, also referred to as parameters, in the Phred algorithm is limited.

Thus each complete base-calling software consists of two parts: base-calling and quality score definition. Bustard is the base-caller developed by Illumina/Solexa and is the default method embedded in the Illumina sequencers. Its base-calling module includes image processing, extraction of cluster intensity signals, corrections of phasing and color crosstalk, normalization etc. Its quality scoring module generates error rates using a modification of the Phred algorithm, namely, a lookup table method, on a calibration data set. The Illumina quality scoring system was briefly explained in its manual [4] without details. Recently, we developed a new base-caller 3Dec [5], whose preprocessing further carries out adaptive corrections of spatial crosstalks between neighboring clusters. Compared to other existing methods, it reduces the error rate by 44-69%. However, the model to predict quality scores has not been fully investigated yet.

**Fig. 1:**
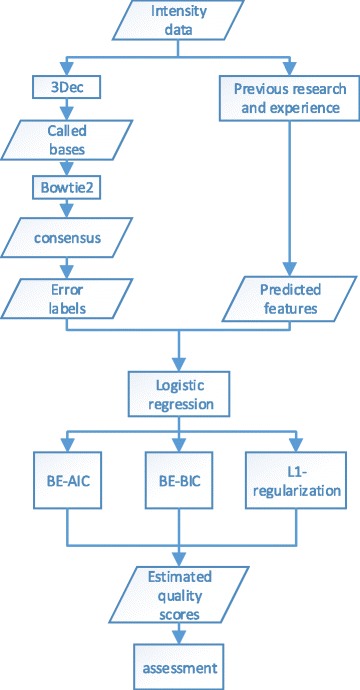
The flowchart of the method. The input is the raw intensities from sequencing. Then the called sequences are obtained using 3Dec. Next we used Bowtie2 to map the reads to the reference and defined a consensus sequence. Thus bases that are called different from those in the consensus reference are regarded as base-calling errors. Meanwhile a group of predictive features are calculated from the intensity data followed previous research and experience. Afterwards, three sparse constrained logistic regressions are carried out, and they are backward deletion either with BIC(BE-BIC) and AIC(BE-AIC), and *L*_1_-regularization respectively. Finally, we use several measures to assess the predicted quality scores of the above three methods

In this paper, we evaluate the error probabilities of base calls from predictive features of sequencing signals, using logistic regression models [6]. The basic idea of the method is illustrated in Fig. 1. Logistic regression, as one of the most important classes of generalized linear models [7], is widely used in statistics for evaluating success rate of binary data from dependent variables and in machine learning for classification problems. The training of logistic regression models can be implemented by the well-developed maximum likelihood method, and is computed by Newton-Raphson algorithm [8]. Instead of restricting to a limited number of experimental features, we include a large number of candidate features in our model and select predictive features via the sparse modeling. From previous research [9, 10] and our recent work (3Dec [5]), the candidate features for Illumina sequencing platforms should include: signals after correction for color-, cyclic- and spatial-crosstalk, the cycle number of the current positions, the two most likely nucleotide bases of the current positions and the called bases of the neighbor positions. In this article, we select 74 features derived from these factors as the predictive variables in the initial model.

Next, we reduce the initial model by imposing sparsity constraints. That is, we impose a *L*_0_ or *L*_1_ penalty on the log-likelihood function of the logistic models, and optimize the penalized function. The *L*_0_ penalty includes the Akaike information criterion (AIC) and Bayesian information criterion (BIC). However, the exhaustive search of minimum AIC or BIC in all sub-models is a NP-hard problem [11]. An approximate solution can be achieved by the backward deletion strategy, whose computational complexity is polynomial. We note that this strategy coupled with BIC leads to the consistent model estimates in the case of linear regression [12]. Thus it is hypothesized that the same strategy would lead to a similar consistent asymptotics in the case of logistic regressions. Compared to BIC, AIC is more appropriate in finding the best model for predicting future observations [13]. The *L*_1_ regularization, also known as LASSO [14], has recently become a popular tool for feature selection. Its solution can be solved by fast convex optimization algorithms [15]. In this article, we use these three methods to select the most relevant features from the initial ones.

In fact, a logistic model was already used to calibrate the quality values of training data sets that may come from different experiment conditions [16]. The covariates in the logistic model are simple spline functions of original quality scores. The backward deletion strategy coupled with BIC was used to pick up the relevant knots. In the same article, the accuracy of quality scores was examined by the consistency between empirical (aka. observed) error rates and the predicted ones. Besides, the scoring method could be measured by the discrimination power, namely, the ability to discriminate the more accurate base-calls from the less accurate ones. Ewing et al. [2] demonstrated that the bases of high quality scores are more important in the downstream analysis such as deriving the consensus sequence. Technically, they defined the discrimination power as the largest proportion of bases whose expected error rate is less than a given threshold. However, this definition is not perfect if bias exists, to some extent, in the predicted quality scores of a specific data set. Thus, in this article, we propose an empirical version of discrimination power, which is used for comparing the proposed scoring method with that of Illumina.

The sparse modeling using logistic regressions not only defines valid Phred scores, but also provides insights into the error mechanism of the sequencing technology by variable selection. Like the AIC and BIC method, the solution to *L*_1_-regularized method is sparse and thereby embeds variable selection. The features identified by the model selection are good explanatory variables that may even lead to the discoveries of causal factors. For example, quenching effect [17] is a factor leading to uneven fluorescence signals, due to short-range interactions between the fluorophore and the nearby molecules. Using the logistic regression methods, we further demonstrated the detailed pattern of G-quenching effect in the Illumina sequencing technology, including G-specific phasing and the reduction of the T-signal following a G.

## Methods

### Data

The source data used in this article were from [18], and were downloaded at [19]. This dataset includes three tiles of raw sequence intensities from Illumina HiSeq 2000 sequencer. Each tile contains about 1,900,000 single-end reads of 101 sequencing cycles, whose intensities are from four channels, namely A, C, G and T. Then we carried out the base calling using 3Dec [5] and obtained the error labels of the called bases by mapping the reads to the consensus sequence. The more than 400X depths of sequencing reads make it possible to define a reliable consensus sequence, and the procedure is the same as [5]. That is, first, Bowtie2 (version 2.2.5, using the default option of “ −sensitive”) was used to map the reads to the reference (Bacteriophage PhiX174). Second, in the resulting layout of reads, a new consensus was defined as the most frequent nucleotide at each base position. Finally, this consensus sequence was taken as the updated reference. According to this scheme, the bases that were called different from those in the consensus reference were regarded as the base-calling errors. In this way, we obtained the error labels of the called bases. We selected approximately three million bases of 30 thousand sequences from the first tile as the training set, and tested our methods on a set of bases from the third tile.

Throughout the article, we represent random variables by capital letters, their observations by lowercase ones, and vectors by bold ones. We denote the series of target bases in the training set by *S*=*S*_1_ *S*_2_⋯*S*_*n*_, where *S*_*i*_ is the called base taking any value from the nucleotides A, C, G or T. Let *Y*_*i*_ be the error label of base *S*_*i*_ (*i*=1,2,⋯,*n*). Therefore,

$$Y_{i}= \left \{ \begin{array}{ll} 1 & \text{if \,base\, \(S_{i}\)\, is\, called\, correctly\,,}\\ 0 & \text{otherwise\,.} \\ \end{array} \right. $$

### Phred scores

Many existing base-calling software output a quality score *q* for each base call to measure the error probability after the influential work of Phred scores [2]. Mathematically, let *q*_*i*_ be the quality score of the base *S*_*i*_, then

1 $$ \left \{ \begin{array}{lll} q_{i}& = & -10\log_{10}\varepsilon_{i} \,,\\ \varepsilon_{i}& = & \mathbf{Pr}(Y_{i} = 0|\boldsymbol{X}_{i}=\boldsymbol{x}_{i}) \,,\\ \end{array} \right.  $$

where *ε*_*i*_ is the error probability of base-calling and *X*_*i*_ is the feature vector described below. For example, if the Phred quality score of a base is 30, the probability that this base is called incorrectly is 0.001. This also indicates that the base call accuracy is 99.9%. The estimation of Phred scores is equivalent to the estimation of the error probabilities.

### Logistic regression model

Ewing et al. proposed the lookup table stratified by four features to predict quality scores [2]. Here, we adopt a different stratification strategy using the logistic regression [6].

Mathematically, the logistic regression model here estimates the probability that a base is called correctly. We denote this probability for the base *S*_*i*_ as

2 $$ p(\boldsymbol{x}_{i}; \boldsymbol\beta) = 1- \varepsilon_{i} = \mathbf{Pr}(Y_{i}=1|\boldsymbol{X}_{i}=\boldsymbol{x}_{i};\boldsymbol\beta),  $$

where ***β*** is the parameter to be estimated. We assume that *p*(***x***_*i*_;***β***) follows a logistic form:

3 $$ \log \left(\frac{p(\boldsymbol{x}_{i}; \boldsymbol\beta)}{1-p(\boldsymbol{x}_{i}; \boldsymbol\beta)} \right) = \boldsymbol{x}_{i}^{T} \boldsymbol\beta,  $$

where the first element in ***x***_*i*_ is a constant, representing the intercept term. Equivalently, the accuracy of base-calling can be represented as:

4 $$ p(\boldsymbol{x}_{i}; \boldsymbol\beta) = \frac {1} {1+ \exp\left(-\boldsymbol{x}_{i}^{T} \boldsymbol\beta\right)}.  $$

The above parameterization leads to the following form of log-likelihood function for the data of base calls:

5 $$ \begin{aligned} L(\boldsymbol\beta;\boldsymbol{x}_{1},\cdots,\boldsymbol{x}_{n})&=\sum\limits_{i=1}^{n} \left(y_{i} \log p(\boldsymbol{x}_{i};\boldsymbol\beta) + (1-y_{i})\right.\\&\quad\left.\log (1-p(\boldsymbol{x}_{i};\boldsymbol\beta))\right), \end{aligned}  $$

where *y*_*i*_ is the value of *Y*_*i*_, namely 0 or 1, and ***β*** represents all the unknown parameters. Then ***β*** is estimated by maximizing the log-likelihood function, and is computed by the Newton-Raphson algorithm [8].

The computation of logistic regression is implemented by the “glm” package provided in the R software [20], in which we take the parameter “family” as binomial and take the “link function” as the logit function.

### Predictive features of Phred scores

Due to the complexity of the lookup table strategy, the number of predictive features in the Phred algorithm is limited for Sanger sequencing reads. Ewing et al. [2] used only four trace features such as peaking spacing, uncalled/called ratio and peak resolution to discriminate errors from correct base-calls [2]. However, these features are specific in the Sanger sequencing technology and are no longer suitable for next generation sequencers. In addition, next generation sequencing methods have their own error mechanism leading to incorrect base-calls such as the phasing effect. From previous research [9, 10] and our recent work [5], it should be noted that the error rates of the base calls in the Illumina platforms are related to the factors such as the signals after correction for color-, cyclic- and spatial crosstalk, the cycle number of current positions, the two most likely nucleotide bases of current positions and the called bases of the neighbor positions. Therefore, a total of 74 candidate features are included as the predictive variables in the initial model. Let ***X***_*i*_=(*X*_*i*,0_,*X*_*i*,1_,⋯,*X*_*i*,74_) be the vector of the predictive features for the base *S*_*i*_, and we explain them in groups as follows. Notice that some features are trimmed off to reduce their statistical influence of outliers.

- *X*_*i*,0_ equals 1, representing the intercept term.
- *X*_*i*,1_, *X*_*i*,2_ are the largest and second largest intensities in the *i* ^*th*^ cycle, respectively. Because 3Dec [5] assigns the called base of *i* ^*th*^ cycle as the type with the largest intensity, the signal intensities such as *X*_*i*,1_ and *X*_*i*,2_ are crucial to the estimation of error probability. It makes sense that the called base is more accurate if *X*_*i*,1_ is larger. On the contrary, the called base *S*_*i*_ has a tendency to be miscalled if *X*_*i*,2_ is large as well, because the base calling software may be confused to determine the base with two similar intensities.
- *X*_*i*,3_, *X*_*i*,4_ and *X*_*i*,5_ are the average of *X*_*i*,1_, the average and standard error of |*X*_*i*,1_−*X*_*i*,2_| in all the cycles in that sequence, respectively. The average signals outside [0.02, 3] and the standard error outside [0.02, 1] were trimmed off. *X*_*i*,3_ to *X*_*i*,5_ are common statistics that describe the intensities over the whole sequence.
- *X*_*i*,6_,*X*_*i*,7_,*X*_*i*,8_ are 1/*X*_*i*,3_, $\sqrt {X_{i,5}}$ and log(*X*_*i*,5_), respectively. *X*_*i*,9_ to *X*_*i*,17_ are nine different piecewise linear functions of |*X*_*i*,1_−*X*_*i*,2_|, which are similar to [16]. *X*_*i*,6_ to *X*_*i*,17_ are used to approximate the potential non-linear effects of the former features.
- *X*_*i*,18_ equals the current cycle number *i*, and *X*_*i*,19_ is the inverse of the distance between the current and last cycle. These two features are derived from the position in the sequence due to the facts that bases close to both ends of sequences are more likely to be miscalled [18].
- *X*_*i*,20_ to *X*_*i*,26_ are seven dummy variables [21], each representing whether the current cycle *i* is the first, the second, …, the seventh, respectively. We add these seven features because the error rates in the first seven cycles of this dataset are fairly high [18].
- *X*_*i*,27_ to *X*_*i*,74_ are 48 dummy variables, each representing a 3-letter-sequence. The first letter indicates the called base in the previous cycle; the second and third letter respectively correspond to the nucleotide type with the largest and the second largest intensity in the current cycle. It is worth noting that these 3-letter-seuqnces involve only two DNA neighbor positions, instead of three. Take “A(AC)” as an example, the first letter “A” indicates the called base of the previous cycle, namely *S*_*i*−1_; the second letter “A” in the parenthesis represents the called base in the current cycle, namely *S*_*i*_; and the third letter “C” in the parenthesis is corresponding to the nucleotide type with the second largest intensity in the current cycle. All the 48 possible combinations of such 3-letter sequences are sorted in lexicographical order, which are “A(CA)”, “A(CG)”, “A(CT)”, …, “T(GT)”, respectively.
  The 48 features are chosen based on the facts that the error rate of a base varies when preceded by different bases [10]. These 3-letter sequences derived from the two neighboring bases can help us understand the differences among the error rates of the bases preceded by “A”, “C”, “G” and “T”. Back to the example mentioned earlier, if the coefficient of “A(AC)” was positive, in other word, the presence of “A(AC)” led to a higher quality score, we would consider that an “A” after another “A” was more likely to be called correctly. On the contrary, if the coefficient of “A(AC)” was negative, the presence of “A(AC)” would reduce the quality score. In this case, there would be a high probability that the second “A” was an error while the correct one was “C”. Thus it would indicate a substitution error pattern between “A” and “C” proceeded by base “A”.

### Sparse modeling and model selection

To avoid overfitting and to select a subset of significant features, we reduce the initial logistic regression model by imposing sparsity constraints. That is, we impose a *L*_0_ or *L*_1_ penalty to the log-likelihood function of the logistic models, and optimize the penalized function.

The *L*_0_ penalty includes AIC and BIC, which are respectively defined as

6 $$\begin{array}{*{20}l} AIC &= 2k -2\hat{L}, \end{array} $$

7 $$\begin{array}{*{20}l} BIC &= k\log(n) - 2\hat{L}, \end{array} $$

where *k* is the number of non-zero parameters in the trained model referred to as ||***β***||_0_, *n* is the number of samples, and $\hat {L}$ is the maximum of the log-likelihood function defined in Eq. (5). AIC and BIC look for a tradeoff between the goodness of fit (the log-likelihood function) and the model complexity (the number of parameters). The smaller the AIC/BIC score is, the better the model is. The exhaustive search of minimum of AIC or BIC among all sub-models is a NP-hard problem [11], thus approximate approaches such as backward deletion are usually used in practice. The computational complexity of the backward deletion strategy is only polynomial. In fact, we note that this strategy coupled with BIC leads to the consistent model estimates in the case of linear regression [12]. Thus it is hypothesized that the same strategy would lead to a similar consistent asymptotics in the case of logistic regressions. Compared to BIC, AIC is more appropriate in finding the best model for predicting future observations [13].

The details of backward deletion are as follows. First, we implement the logistic regression with all features and calculate the AIC and BIC scores. Second, we remove each feature, recalculate the logistic regression models as well as their AIC and BIC scores, then delete the feature resulting in the lowest AIC or BIC score if it was removed. Last, we repeat the second step in the remaining features until AIC or BIC score no longer decreases. We note that this heuristic algorithm is still very time consuming due to the repetitive calculation of the logistic regression.

An alternative approach for sparse modeling is *L*_1_ regularization. It imposes a *L*_1_ norm penalty on the objective function, rather than the hard constraint on the number of nonzero parameters. Specifically, *L*_1_-regularized logistic regression is to minimize the log-likelihood function penalized by the *L*_1_ norm penalty of the parameters as follows:

8 $$ \min_{\boldsymbol\beta} -L(\boldsymbol\beta;\boldsymbol{x}_{1},\cdots,\boldsymbol{x}_{n}) + \lambda ||\boldsymbol\beta||_{1},  $$

where ||***β***||_1_ is the sum of the absolute value of each element in ***β***, and *λ* is specified based on a certain cross-validation procedure. The *L*_1_ regularization, also known as LASSO [14], is applied here due to its two merits: first, it leads to a convex optimization problem which is well studied and can be solved very fast; second, it often produces a sparse solution which embeds feature selection and enables model interpretation. We further extended LASSO to the elastic net model [22], and the details were described in Additional file 1.

All these three methods seek for a tradeoff between the goodness of fit and model complexity. They also extract underlying sparse patterns from high dimensional features to enhance the model interpretability. However, they may result in different sparse solutions. If the data size *n* is large enough, log(*n*) is much larger than 2, then backward deletion with BIC results in a sparser result than the AIC procedure does. Similarly, the sparsity of *L*_1_ regularization depends on *λ*. The larger *λ* is, the sparser the solution is.

The backward deletion with either AIC or BIC is implemented by the “stepAIC” function in “MASS” package provided in R [20], and *L*_1_-regularized logistic regression is implemented in C++ using the liblinear library [23].

### Model assessment

#### Consistency between predictive and empirical error rates

First, we follow Ewing et al. [2] and Li et. al. [16] to calculate the observed score stratified by the predicted ones. The observed score for the predicted quality score *q* is calculated by

9 $$ q_{obs}(q) = -10\cdot\log_{10}\left(\frac{Err_{q}}{Err_{q} + Corr_{q}}\right),  $$

where *Err*_*q*_ and *Corr*_*q*_ are, respectively, the number of incorrect and correct base-calls at quality score *q*. The consistency between the empirical scores with the predicted ones indicates the accuracy of the model.

#### Empirical discrimination power

Second, Ewing et al. [2] proposed that the quality scores could be evaluated by the discrimination power, which is the ability to discriminate the more accurate base-calls from the less accurate ones.

Let *B* be a set of base-calls and *e*(*b*) be the error probability assigned by a valid method for each called base *b*. For any given error rate *r*, there exists a unique largest set of base-calls, *B*_*r*_, satisfying two properties: (1) the expected error rate of *B*_*r*_, i.e. the average assigned error probabilities of *B*_*r*_ is less than *r*; (2) whenever *B*_*r*_ includes a base-call *b*, it includes all other base-calls whose error probabilities are less than *e*(*b*). The discrimination power at the error rate *r* is defined as

$$\begin{array}{*{20}l} P_{r} = \frac{|B_{r}|}{|B|}. \end{array} $$

However, if bias exists, to some extent, in the predicted quality scores of a specific data set, the above definition is not perfectly fair. For example, if an inconsistent method assigns each base call a fairly large score, then *P*_*r*_ reaches 1 at any *r*. Therefore, its discrimination power is much larger than any consistent method, which is obviously unfair. Thus we proposed an empirical version of discrimination power, defined as:

10 $$ \widetilde{P_{r}} = \frac{|\widetilde{B_{r}}|}{|B|},  $$

where the above *B*_*r*_ is replaced by $\widetilde {B_{r}}$ having the properties that: ($\widetilde {1}$) the empirical error rate of $\widetilde {B_{r}}$, i.e. the number of errors divided by the number of base-calls in $\widetilde {B_{r}}$, is less than *r*. (2) the same as that in Ewing et al’s definition, see above. When little bias exists in the estimated error rates, the empirical discrimination power converges to the one proposed in Ewing et al. [2].

We note that the calculation of empirical discrimination power requires the information of base call errors, which could be obtained by mapping reads to a reference. Then $\widetilde {P_{r}}$ is calculated as follows: (1) sort the bases in descending order by their predicted quality scores; (2) for each base, generate a set containing the bases from the beginning to the current one, and calculate its empirical error rate; (3) for a given error rate *r*, select the largest set whose empirical error rate is less than *r*; (4) $\widetilde {P_{r}}$ equals the number of base calls in the selected set divided by the number of total bases.

We can take the quality scores as reliability measures of base-calls, assuming that the bases with higher scores are more accurate. Therefore, a higher $\widetilde {P_{r}}$ indicates that a method could identify more reliable bases for a given empirical error rate. By plotting the empirical discrimination power versus the empirical error rate *r*, we can compare the performance of different methods.

#### ROC curve

Last, we plot the ROC and Precision-Recall curve to compare the methods. That is, by adjusting various quality score thresholds, we can classify the bases as correct and incorrect calls based on their estimated scores, and calculate the true positive rate against false positive rate and plot the ROC curve [24]. The area under the ROC curve (AUC) represents the probability that the quality score of a randomly chosen correctly called base is larger than the score of a randomly chosen incorrectly one.

## Results and discussion

### Model training

First we trained the model by the AIC, BIC and *L*_1_ regularization method using a data set of about 3 million bases from a tile. The computation was implemented on a Dell T7500 workstation that has an Intel Xeon E5645 CPU and 192 GB RAM. It took about 50 h to train the model using the backward deletion coupled with either AIC or BIC. In comparison, the *L*_1_ regularization training took about 2 min only. As we increased the size of training data set to 5- and 50-folds, the workstation could no longer finish the training by the AIC or BIC method in a reasonable period of time while it respectively took 5 and 15 min for the *L*_1_ regularization training.

The coefficients of the trained model using a data set of 3 million bases are shown in Table 1. If we compare the models trained on the 3 million base dataset, the backward deletion with BIC deleted 53 variables, and the backward deletion with AIC eliminated 14 ones. Besides, the latter is a subset of the former. Unlike AIC/BIC, the sparsity of *L*_1_-regularized logistic regression depends on the parameter *λ*. Here *λ* was chosen by a cross-validation method that maximizes AUC as described in Methods. When we took *λ* to be 1.0, the *L*_1_ regularization removed 11 variables, two of them were not removed by BIC. Overall, The BIC method selected the least number of features, thus was most helpful for model interpretation.

**Table 1:** The coefficients of the 74 predictive variables in the three methods

x	Description	L1LR	BE-AIC	BE-BIC
x0	intercept	1.09	11.47	7.63
x1	largest intensity	1.48	-	-
x2	second largest intensity	-1.73	-4.84	-4.42
x3	average of x1	-1.18	-	-
x4	average of (x1-x2)	-4.65	-6.2	-5.65
x5	standard error of (x1-x2)	3.19	-10.03	-
x6	1/x3	-2.37	-3.22	-2.88
x7	*√*x5	0.54	1.42	0.77
x8	log(x5)	-0.93	2.69	-
x9	piecewise function of |x1-x2|	0.59	-	-
x10		3.53	4.94	4.71
x11		3.45	6.62	6.3
x12		2.42	9.32	8.74
x13		1.44	12.35	11.43
x14		0.34	15.41	14.21
x15		-	23.06	21.45
x16		-	118.87	46.79
x17		-	-	-
x18	current cycle number	-0.016	-0.019	-0.018
x19	inverse distance	-0.24	-	-
x20	indicators of the first 7th cycles	-0.3	-2.99	-
x21		-0.15	-	-
x22		-	-	-
x23		-	-	-
x24		-0.25	-	-
x25		-0.54	-1.22	-
x26		0.32	12.49	-
x27	A(AC)	-0.11	-	-
x28	A(AG)	-0.91	-2.21	-1.32
x29	A(AT)	-0.67	-3.39	-1.15
x30	A(CA)	1.29	-	-
x31	A(CG)	0.86	-2.89	-
x32	A(CT)	0.25	-5.31	-
x33	A(GA)	1.44	-3.23	-
x34	A(GC)	0.21	-5.8	-
x35	A(GT)	1.66	-6.51	-
x36	A(TA)	0.89	-6.96	-
x37	A(TC)	0.44	-8.77	-
x38	A(TG)	-	-10.79	-
x39	C(AC)	2.27	2.88	2.29
x40	C(AG)	-	-1.34	-
x41	C(AT)	-	-2.77	-
x42	C(CA)	-0.95	-2.65	-1.4
x43	C(CG)	-0.7	-5.29	-
x44	C(CT)	-0.7	-5.29	-
x45	C(GA)	-1.29	-7.09	-1.68
x46	C(GC)	0.89	-3.51	-
x47	C(GT)	0.63	-5.31	-
x48	C(TA)	0.68	-7.14	-
x49	C(TC)	-	-9.25	-
x50	C(TG)	-0.54	-11.32	-
x51	G(AC)	0.58	-1.09	-
x52	G(AG)	0.05	-1.09	-
x53	G(AT)	-0.45	-3.32	-1.1
x54	G(CA)	0.18	-1.4	-
x55	G(CG)	-0.18	-4.54	-
x56	G(CT)	-1.02	-6.89	-1.52
x57	G(GA)	1.6	-2.78	-
x58	G(GC)	0.24	-5.76	-
x59	G(GT)	-0.75	-9.81	-1.28
x60	G(TA)	0.93	-7.26	-
x61	G(TC)	0.24	-9.18	-
x62	G(TG)	0.7	-10.12	-
x63	T(AC)	-	-	-
x64	T(AG)	-0.23	-1.68	-
x65	T(AT)	2.03	-	-
x66	T(CA)	0.21	-1.28	-
x67	T(CG)	-0.74	-5.27	-
x68	T(CT)	-0.1	-5.72	-
x69	T(GA)	0.16	-4.64	-
x70	T(GC)	0.73	-5.15	-
x71	T(GT)	1.94	-6.55	-
x72	T(TA)	-	-8.09	-
x73	T(TC)	-0.29	-9.76	-
x74	T(TG)	-0.99	-11.72	-

We denote these 74 variables by *x*=(*x*_0_,*x*_1_,⋯,*x*_74_). In the first row of the table, ‘L1LR’ means the *L*_1_-regularized logistic regression, ‘BE-AIC’ indicates the backward deletion with AIC, and ‘BE-BIC’ represents the backward deletion with BIC. The details of the variables in each row are described in Methods. *x*_27_ to *x*_74_ are corresponding to the 3-letter sequences, which indicate the type of the base in the previous cycle, type of the base with the largest and the second largest intensity in current cycle. Meanwhile, ‘-’ implies that the method has removed the feature

We also calculated the contribution of each feature, defined as the t-score, namely, the coefficient divided by its standard error. As shown in Table 2, we listed the contribution of each feature, and classified the features into different groups by the method it was selected. The features contributing the most to all three methods were *x*_10_ to *x*_14_, which were the transformations of *x*_1_−*x*_2_, namely the difference between the largest and second largest intensities. It makes sense that the model could discriminate called-bases more accurate if the largest intensity is much larger than the second largest intensity.

**Table 2:** The contribution of each feature in the three methods: the backward deletion with either AIC or BIC and the *L*_1_ regularization method

	Selected methods		Contribution		
	L1 & AIC & BIC	Description	L1	AIC	BIC
1	x2	second largest intensity	-7.2243	-20.211	-18.458
2	x4	average of (x1-x2)	-21.93	-29.24	-26.646
3	x6	1/x3	-8.696	-11.815	-10.567
4	x7	*√*x5	0.47013	1.2363	0.67037
5	x10	piecewise function of |x1-x2|	35.389	49.525	47.219
6	x11		14.579	27.974	26.622
7	x12		7.4602	28.731	26.943
8	x13		4.3013	36.89	34.142
9	x14		2.3397	106.05	97.787
10	x18	current cycle number	-0.00054878	-0.00065167	-0.00061738
11	x28	A(AG)	-5.3348	-12.956	-7.7384
12	x29	A(AT)	-4.2171	-21.337	-7.2382
13	x39	C(AC)	14.771	18.74	14.901
14	x42	C(CA)	-8.0916	-22.571	-11.925
15	x45	C(GA)	-10.411	-57.22	-13.558
16	x53	G(AT)	-3.3127	-24.44	-8.0976
17	x56	G(CT)	-7.7223	-52.163	-11.508
18	x59	G(GT)	-5.893	-77.08	-10.057
	AIC & BIC	Description	L1	AIC	BIC
1	x15	piecewise function of |x1-x2|	0	859.11	799.13
2	x16		0	19180	7549.6
	L1 & AIC	Description	L1	AIC	BIC
1	x5	standard error of (x1-x2)	121.95	-383.44	0
2	x8	log(x5)	-2.8995	8.3866	0
3	x20	indicators of the first 7th cycles	-3.0296	-30.195	0
4	x25		-5.4533	-12.32	0
5	x26		3.2316	126.13	0
6	x31	A(CG)	5.7108	-19.191	0
7	x32	A(CT)	1.864	-39.591	0
8	x33	A(GA)	9.9989	-22.428	0
9	x34	A(GC)	1.4679	-40.542	0
10	x35	A(GT)	11.712	-45.93	0
11	x36	A(TA)	5.7597	-45.042	0
12	x37	A(TC)	2.8418	-56.643	0
13	x43	C(CG)	-5.6759	-42.894	0
14	x44	C(CT)	-5.8779	-44.42	0
15	x46	C(GC)	7.3038	-28.805	0
16	x47	C(GT)	5.051	-42.573	0
17	x48	C(TA)	4.8103	-50.508	0
18	x50	C(TG)	-4.0949	-85.841	0
19	x51	G(AC)	4.0089	-7.5339	0
20	x52	G(AG)	0.3575	-7.7936	0
21	x54	G(CA)	1.1807	-9.1835	0
22	x55	G(CG)	-1.2149	-30.643	0
23	x57	G(GA)	13.802	-23.981	0
24	x58	G(GC)	1.9919	-47.805	0
25	x60	G(TA)	6.2969	-49.157	0
26	x61	G(TC)	1.9621	-75.049	0
27	x62	G(TG)	5.7278	-82.807	0
28	x64	T(AG)	-1.6306	-11.911	0
29	x66	T(CA)	1.6158	-9.8488	0
30	x67	T(CG)	-5.0538	-35.991	0
31	x68	T(CT)	-0.72808	-41.646	0
32	x69	T(GA)	1.0712	-31.065	0
33	x70	T(GC)	5.0709	-35.774	0
34	x71	T(GT)	12.661	-42.749	0
35	x73	T(TC)	-1.7016	-57.268	0
36	x74	T(TG)	-6.1194	-72.443	0
	AIC	Description	L1	AIC	BIC
1	x38	A(TG)	0	-72.981	0
2	x40	C(AG)	0	-8.7049	0
3	x41	C(AT)	0	-19.167	0
4	x49	C(TC)	0	-63.57	0
5	x72	T(TA)	0	-48.82	0
	L1	Description	L1	AIC	BIC
1	x1	largest intensity	1.0896	0	0
2	x3	average of x1	-6.0558	0	0
3	x9	piecewise function of |x1-x2|	13.791	0	0
4	x19	inverse distance	-2.0626	0	0
5	x21	indicators of the first 7th cycles	-1.5148	0	0
6	x24		-2.5247	0	0
7	x27	A(AC)	-0.59405	0	0
8	x30	A(CA)	11.7	0	0
9	x65	T(AT)	15.749	0	0
	None	Description	L1	AIC	BIC
1	x17	piecewise function of |x1-x2|	0	0	0
2	x22	indicators of the first 7th cycles	0	0	0
3	x23		0	0	0
4	x63	T(AC)	0	0	0

The contribution is defined by the t-score, namely the coefficient divides by its standard error. All 74 features are classified into different groups by the method it is selected

We have defined a consensus sequence described in Methods. This strategy may not eliminate the influence of polymorphism. Polymorphisms do occur in this data set of sequencing reads of Bacteriophage PhiX174, but they are very rare. Generally, we could use variant calling methods, such as GATK-HC [25] and Samtools [26], to identify variants and then remove those bases mapped to the variants. This could be achieved by replacing the corresponding bases in the reference by “N”s before the second mapping (the first mapping is for variant calling). In addition, this proposal has been implemented in the updated training module of 3Dec, which was published in the accompany paper [5].

### Consistency between predictive and empirical error rates

**Fig. 2:**
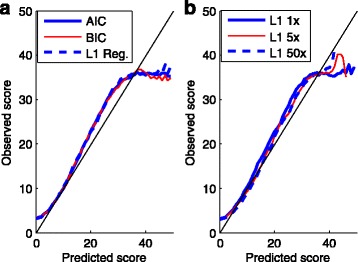
The observed quality scores versus the predicted ones by different methods and by different sizes of training sets. The predicted scores, or equivalently, the predicted error rates of the test dataset were calculated according to the model learned from the training dataset, and the observed (aka. empirical) ones were calculated as -10*log10 [(total mismatches)/(total bp in mapped reads)]. **a** The logistic model for scoring were trained by the three methods: backward deletion with either AIC or BIC, and *L*_1_ regularization using a training data of 3 million bases. **b** The model for scoring were obtained by *L*_1_ regularization with three different training sets, each containing 1-, 5-, and 50-folds of 3 million bases, respectively

We assessed the quality-scoring methods in several aspects. First, following Ewing et al. [2] and Li et al. [16], we assess the consistency of error rates predicted by each model. That is, we plotted the observed scores against the predicted ones obtained from each method. The results from the 3 million base training dataset are shown in Fig. 2. Little bias was observed when the score is below 20. All the three methods slightly overestimate the error rates between 20 and 35, and underestimate the error rates after 35.

The bias decreases as we increased the size of the training dataset to 5- and 50-folds, as shown in Fig. 2. But in these two cases, only *L*_1_ regularization results are available due to the computational complexity. Thus if we expect more accurate estimates of error rates, then we need larger training datasets and the *L*_1_ regularization training is the computational choice.

### Empirical discrimination power

As described in “Methods” section, a good quality scoring method is expected to have high empirical discrimination power, especially in the high quality score range. We calculated the empirical discrimination power for each method, based on the error status of the alignable bases in the test sets.

**Fig. 3:**
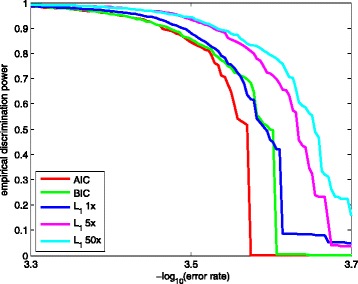
Empirical discrimination powers for three methods: backward deletion with either AIC or BIC, and *L*_1_ regularization. The x-axis is the -log10 (error rate) in the range between 3.3 and 3.7. The y-axis is the empirical discrimination power defined as the largest proportion of bases whose empirical error rate is less than 10^−*x*^. *L*_1_ 1x, 5x, 50x indicates that the *L*_1_-regularized model is trained with 1-, 5-, 50-folds of 3 million bases, respectively

The results are shown in Fig. 3, where the x-aixs is the -log10(error rate) in the range between 3.3 to 3.7, and the y-axis is the empirical discrimination power. If we took the 3 million bases training dataset, the BIC and the *L*_1_ method show comparable discrimination powers, and both outperform the AIC method by around 60% at the error rate 3.58×10^−4^. On average, the empirical discrimination power of the BIC and the *L*_1_ method is 6% higher than that of the AIC method.

Moreover, we compared the empirical discrimination power of the *L*_1_ regularization method with different training sets. As the size of training data goes up, higher empirical discrimination power is achieved at almost any error rate by the *L*_1_ regularization method. The 5-, 50-folds data respectively gains 10 and 14% higher empirical discrimination power than 1-fold data on average. This implies that the *L*_1_ method could identify more highly reliable bases with more training data.

**Fig. 4:**
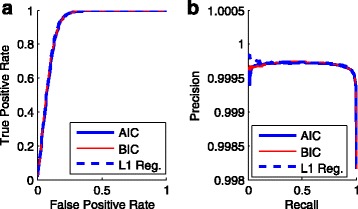
The ROC and Precision-Recall curve for the three methods. A logistic model can be considered as a classifier if we set a threshold to the Phred scores. The predicted condition of a base is positive/negative if its Phred score is larger/smaller than the threshold. The true condition of a base is obtained from the mapping of reads to the reference. Consequently, bases will be divided into four categories: true positives, false positive, true negatives, and false negatives. **a** The ROC curve on the test set by the three methods: the backward deletion with either AIC or BIC, and the *L*_1_ regularization. **b** The corresponding precision-recall curve

We also used the concepts in classification such as the ROC and the Precision-Recall curve to assess the three methods. As shown in Fig. 4, the *L*_1_ regularization achieves the highest precision in the range of high-quality scores, and in most other cases the three methods perform similarly. The AUC scores of the ROC curve for AIC, BIC, and *L*_1_ regularization were 0.9141, 0.9161, and 0.9175, respectively, which show no significant difference.

The detailed results of the elastic net model were described in Additional file 1.

### Comparison with the Illumina scoring method

To be clear, hereafter we refer to the Illumina base-calling as Bustard, and the Illumina quality scoring method as Lookup. Similarly, we refer to the new base calling method [5] as 3Dec and the new quality scoring scheme as Logistic.

In fact, we could exchange the use of Lookup and Logistic with the two base calling methods Bustard and 3Dec. We abbreviate these four schemes by Bustard+Lookup, Bustard+Logistic, 3Dec+Lookup, 3Dec+Logistic respectively, and the details are shown in Table 3. We note that the training of logistic models here involves only *L*_1_ regularization with 100-folds data.

**Table 3:** Four combinations of schemes between two base-calling and two quality scoring methods

		Quality scoring	
		L1-regularized logistic regression	Lookup table strategy
Base-calling	Bustard	Bustard+Logistic	Bustard+Lookup
	3Dec	3Dec+Logistic	3Dec+Lookup

We have two base calling methods: Bustard, the default method embedded in the Illumina sequencers; 3Dec, our newly developed method. We also have two quality scoring methods: Lookup, the lookup table strategy adopted by Illumina; Logistic, the *L*_1_-regularized logistic regression model proposed in this study

To have a systematic comparison of the scoring methods, we need to implement the four schemes in practice. First, Bustard+Lookup is the default method of Illumina. Second, we notice that the definition of quality scores depends on the cluster intensity files but not on the corresponding base calls. We have successfully extracted cluster intensity files preprocessed by Bustard, and input them into the Logistic scoring model. In this way, we implemented Bustard+Logistic.

As to the remaining two schemes, the implementation of 3Dec+Lookup is challenging because it is very hard to separate the quality scoring module from the Illumina systems. As a good approximation, we input the cluster intensity files preprocessed by 3Dec into Bustard, and consequently obtain the quality scores defined by the Phred algorithm provided by Illumina. A subtle issue needs to be explained here. The 3Dec preprocessing of cluster intensity files in fact corrects the spatial crosstalk as well as the phasing and color crosstalk effects. But the Illumina system routinely estimates the effects of phasing and color crosstalk and remove them even if it is unnecessary. Nevertheless, we found this extra step would make little change on the cluster intensity signals. This is supported by the fact: taking the 3Dec preprocessed cluster intensity files as input, the Illumina system outputs base calls highly identical to those by 3Dec. The resulting quality scores are surrogates for those from the 3Dec+Lookup scheme to a good extent. To make a fair comparison, we also use the same cluster intensity signals that have been preprocessed by both 3Dec and Bustard for the Logistic scoring. The resulting quality scores are surrogates for those from the 3Dec+Logistic scheme to a good extent.

**Fig. 5:**
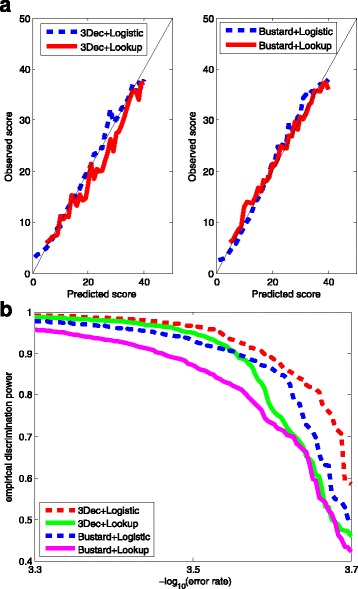
Comparisons of the Logistic quality scoring with the Illumina (Lookup table) scoring method. Two kinds of fluorescence signals preprocessed respectively by 3Dec and Bustard are used for comparisons. There are four combinations of schemes: Bustard+Lookup, Bustard+Logistic, 3Dec+Lookup, and 3Dec+Logistic. The first item is the base-calling method and the second item is the quality scoring method. The detailed implementations of these four schemes are described in Results. We compared them in two aspects: consistency and empirical discrimination power. **a** The consistency of four schemes. *Left*: 3Dec+Logistic and 3Dec+Lookup; *Right*: Bustard+Logistic and Bustard+Lookup. **b** The empirical discrimination powers of four schemes

We compare 3Dec+Logistic versus 3Dec+Lookup from the two aspects: consistency and empirical discrimination power, as shown in Fig. 5. In terms of consistency, by and large, Logistic shows less bias than Lookup does, especially when the scores are less than 25 or between 30 and 40. In terms of discrimination power, Logistic outperforms Lookup across the board. Logistic achieves 25% higher empirical discrimination power at the error rate 3.67×10^−4^, and 6% higher on average than Lookup does.

By the same token, we compare Bustard+Logistic versus Bustard+Lookup, see Fig. 5. In terms of consistency, Logistic shows some bias at the high score end while Lookup shows some bias at the low score end. In terms of discrimination power, Logistic outperforms Lookup by 14% at the error rate 3.62×10^−4^. On average, the empirical discrimination power of Logistic increases by 6% than that of Lookup.

Overall, Logistic defines better quality scores than Lookup does, particularly in the sense that it identifies more base calls of high quality.

### Biological insights

**Fig. 6:**
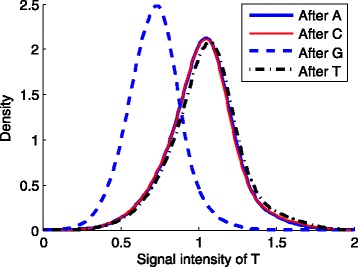
The density plots of “T” signals stratified by the preceding nucleotide bases. First we read the corrected fuorescence signals and the called sequences of the first tile. Then for each nucleotide type X (X=“A”, “C”, “G”, or “T”), we found the sequence fragments “XT” in Cycle 8-12 in all the sequences, and draw the density curves of the signals of “T” in these fragments, respectively. The curve was calculated using the Gaussian kernel with a fixed width of 0.01. As shown in the figure, the signals of “T” preceded by “G” are lower than those after other nucleotide bases

The error patterns identified by the model selection results provide insights into the error mechanism of the sequencing technology. For example, the coefficients of the 3-letter sequences “G(AT)”, “G(CT)”, and “G(GT)” (*x*_53_, *x*_56_, and *x*_59_) are all negative across the three methods. This implies that a nucleotide “T” after a “G” was more likely to be miscalled. To verify this, we plotted the kernel density of fluorescence intensities of “T” stratified by the types of the preceding nucleotide bases. That is, we read the corrected fluorescence signals and the called sequences of the first tile. Then for each nucleotide type X (X=“A”, “C”, “G”, or “T”), we found the sequence fragments “XT” in Cycle 8-12 in all the sequences, and calculated the kernel densities of the signals of “T” in these fragments, respectively. As shown in Fig. 6, the signals of “T” after “G” are lower than those after other types of nucleotide bases. One factor that causes uneven fluorescence signals is the quenching effect [17], due to short-range interactions between the fluorophore and the nearby molecules. The G-quenching factor was included in the quality score definition of the Illumina base-calling [4]. In comparison, our sparse modeling of logistic regression suggested that the most prominent quenching pattern in the current chemistry of Illumina occurred at the dinucleotide “GT”.

Phasing is a phenomenon specific to the technique of reversible terminators. In the presence of phasing, a nucleotide has a larger chance to be miscalled as the preceding one. Interestingly, we found that in the BIC model, only 7 coefficients are negative, of which 5 are corresponding to the pattern “X(XY)” (*x*_28_, *x*_29_, *x*_42_, *x*_44_, and *x*_59_). Similarly, we noticed that in the AIC and *L*_1_ regularization model, most coefficients of the pattern “X(XY)” are non-positive, except those when “X” represents “G”. This implies that nucleotides were more likely to be miscalled as the previous bases if the preceding ones were not “G”. It suggested that the phasing effect of bases after “G” was somewhat different from those after other nucleotide types.

## Conclusions

In the recent years, next-generation sequencing technology has been greatly developed. However, the errors in the both ends of reads are still very high, and low quality called bases result in missing or wrong alignments that strongly affect downstream analysis [27]. So a valid and accurate method to estimate the quality scores is still essential and indispensable. In this article, we applied logistic regression and sparse modeling to predict the quality scores for Illumina sequencing technology. Both the Phred algorithm and our method belong to the supervised learning, since the labels of base-calling errors are obtained from sequence alignment results. Meanwhile, our method has some distinct merits that we explain as follows.

**Fig. 7:**
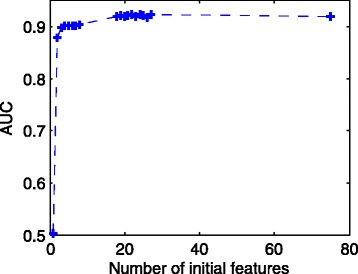
The AUC of the ROC curve versus the number of features in the initial model. In the logistic model, we sequentially include one more feature starting from *x*_0_ to *x*_74_ in Table 1 (the number of features is shown by the x-axis), and calculated each AUC (shown by the y-axis) using the *L*_1_-regularized method

First, the logistic model can take many relevant features. As shown in Fig. 7, the AUC of the *L*_1_ method increases monotonically as we put more features in the model. Therefore, any features that are thought to be associated with the error rates could be included in the initial model. The possible overfitting problem is then overcome by the *L*_0_ or *L*_1_ regularization.

Second, the *L*_1_-regularized logistic regression can be solved in a short period of time, and it has improved performance with more training data. Thus it can handle large dataset and is efficient enough for daily sequencing. Compared to the *L*_1_ method, backward deletion with either AIC or BIC takes a long training time, and it fails to complete the training in a reasonable period of time for the 50-folds dataset. However, the BIC method selects the least number of features, which greatly helps for model interpretation.

Third, our method can be easily modified to adjust other base callers. The features we used are not software-specific. As shown in Fig. 5, the *L*_1_ scoring method outperforms the Illumina scoring method by a great margin in terms of the empirical discrimination power, based on the fluorescence signals preprocessed either by 3Dec or by Bustard. We note that the Illumina system does not have an option that allows us to train it based on the same dataset used by the Logistic method. In conclusion, we recommend the logistic regression with *L*_1_ regularization method to estimate the quality scores.

Fourth, the sparse modeling also helps us discover error patterns that help the downstream analysis. One important application of the sequencing technology is SNP calling. Our results indicate that not only allele frequencies, but also sequencing error patterns can help improve the SNP calling accuracy. Using the logistic regression methods, we further demonstrated the detailed pattern of G-quenching effect including G-specific phasing and the reduction of the T-signal following a G. Therefore, one should take the preceding bases into consideration when performing SNP calling.

Finally, the proposed training method is applicable to sequencing data from any sequencing technique. Meanwhile the resulting model including predictive features and error patterns is specific to the corresponding sequencing technique such as Illumina. Furthermore, the training method is adaptive to the experimental conditions.

## Additional file

Additional file 1 Supplementary information about the elastic net model. This file contains the following sections: **S1** - Introduction to the elastic net model and its advantages. **S2** - Results of the elastic net mode include training time, coefficients, consistency and empirical discrimination power. **Table S1** - The coefficients of 74 predicted features of the elastic net model. **Figure S1** - The consistency of the elastic net model with three different training sets. **Figure S2** - The empirical discrimination power of the elastic net model with three different training sets. (PDF 164 kb)

## Abbreviations

AIC: Akaike information criterion
AUC: area under the curve
BE-AIC: Backward deletion with AIC
BE-BIC: Backward deletion with BIC
BIC: Bayesian information criterion
L1LR: *L*_1_-regularized logistic regression
LASSO: Least absolute shrinkage and selection operator
Logistic: the *L*_1_-regularized logistic regression model
Lookup: the lookup table strategy
ROC: Receiver operating characteristic
SNP: Single nucleotide polymorphism

## References

[1] Mardis ER (2008). Next-generation dna sequencing methods. Annu Rev Genomics Hum Genet.

[2] Ewing B, Green P (1998). Base-calling of automated sequencer traces using Phred. ii. error probabilities. Genome Res.

[3] Bokulich NA, Subramanian S, Faith JJ, Gevers D, Gordon JI, Knight RT, Mills DA, Caporaso JG (2013). Quality-filtering vastly improves diversity estimates from Illumina amplicon sequencing. Nat Methods.

[4] HCS 1.4/RTA 1.12 Theory of Operation. Illumina Inc. http://www.illumina.com/Documents/products/technotes/technote_rta_theory_operations.pdf. Accessed 20 July 2016.

[5] Wang B, Wan L, Wang A, Li LM (2017). An adaptive decorrelation method removes Illumina DNA base-calling errors caused by crosstalk between adjacent clusters. Sci Rep.

[6] Hosmer Jr DW, Lemeshow S (2004). Applied Logistic Regression.

[7] Mccullagh P, Nelder JA (1989). Generalized Linear Models. vol. 37..

[8] Ypma TJ (1995). Historical development of the Newton-Raphson method. SIAM Rev.

[9] Dohm JC, Lottaz C, Borodina T, Himmelbauer H (2008). Substantial biases in ultra-short read data sets from high-throughput dna sequencing. Nucleic Acids Res.

[10] Minoche AE, Dohm JC, Himmelbauer H (2011). Evaluation of genomic high-throughput sequencing data generated on Illumina hiseq and genome analyzer systems. Genome Biol.

[11] Rish I, Grabarnik G (2014). Sparse Modeling: Theory, Algorithms, and Applications.

[12] An H, Gu L (1985). On the selection of regression variables. Acta Math Applicatae Sin.

[13] Chakrabarti A, Ghosh JK (2011). AIC, BIC, and recent advances in model selection. Handbook of the philosophy of science.

[14] Tibshirani RJ (1996). Regression shrinkage and selection via the lasso. J R Stat Soc.

[15] Friedman J, Hastie T, Tibshirani R (2010). Regularization paths for generalized linear models via coordinate descent. J Stat Softw.

[16] Li M, Nordborg M, Li LM (2004). Adjust quality scores from alignment and improve sequencing accuracy. Nucleic Acids Res.

[17] Seidel CAM, And AS, Sauer MHM (1996). Nucleobase-specific quenching of fluorescent dyes. 1. nucleobase one-electron redox potentials and their correlation with static and dynamic quenching efficiencies. J Phys Chem.

[18] Ye C, Hsiao C, Corrada BH (2014). Blindcall: ultra-fast base-calling of high-throughput sequencing data by blind deconvolution. Bioinformatics.

[19] Bravo HC. Research Webpage. http://www.cbcb.umd.edu/%7Ehcorrada/secgen. Accessed 20 July 2016.

[20] R Core Team (2017). R: A Language and Environment for Statistical Computing.

[21] Mcclave JT, Sincich T (2000). Statistics.

[22] Zou H, Hastie T (2005). Regularization and variable selection via the elastic net. J R Stat Soc Ser B Stat Methodol.

[23] Fan RE, Chang KW, Hsieh CJ, Wang XR, Lin CJ (2010). Liblinear: A library for large linear classification. J Mach Learn Res.

[24] Hanley JA, Mcneil BJ (1982). The meaning and use of the area under a receiver operating characteristic (roc) curve. Radiology.

[25] McKenna A, Hanna M, Banks E, Sivachenko A, Cibulskis K, Kernytsky A, Garimella K, Altshuler D, Gabriel S, Daly M, DePristo MA (2010). The Genome Analysis Toolkit: A MapReduce framework for analyzing next-generation DNA sequencing data. Genome Res.

[26] Li H, Handsaker B, Wysoker A, Fennell T, Ruan J, Homer N, Marth G, Abecasis G, Durbin R (2009). The Sequence Alignment/Map format and SAMtools. Bioinformatics.

[27] Del Fabbro C, Scalabrin S, Morgante M, Giorgi FM (2013). An extensive evaluation of read trimming effects on illumina NGS data analysis. PLoS ONE.

