# Supplementary material for: Estimating Phred scores of Illumina base calls by logistic regression and sparse modeling
Source: BMC Bioinformatics. 2017 Jul 11;18:335. doi: 10.1186/s12859-017-1743-4 (PMC5504792; doi:10.1186/s12859-017-1743-4)
Supplement: Additional file 1 — Supplementary information about the elastic net model. This file contains the following sections: S1 - Introduction to the elastic net model and its advantages. S2 - Results of the elastic net mode include training time, coefficients, consistency and empirical discrimination power. Table S1 - The coefficients of 74 predicted features of the elastic net model. Figure S1 - The consistency of the elastic net model with three different training sets. Figure S2 - The empirical discrimination power of the elastic net model with three different training sets. (PDF 164 kb) [file 12859_2017_1743_MOESM1_ESM.pdf]

Supplementary Text for: Estimating Phred Scores of Illumina Base Calls by Logistic Regression and Sparse Modeling

Sheng Zhang, Bo Wang, Lin Wan and Lei M Li

S1 Induction to the elastic net model

The elastic net [1] is a generalization of LASSO [2] and ridge regression [3], which does continuously shrinkage and automatic variable selection simultaneously. Mathematically, the logistic regression with the elastic net regularization is to minimize the negative log-likelihood penalized by the linear combination of the L_1 and L_2 penalties as follows:

$$\min_{\boldsymbol{\beta}} -L(\boldsymbol{\beta}; \boldsymbol{x}_1, \cdots, \boldsymbol{x}_n) + \lambda(\alpha||\boldsymbol{\beta}||_1 + (1-\alpha)||\boldsymbol{\beta}||_2), \tag{1}$$

where $||\boldsymbol{\beta}||_1$ and $||\boldsymbol{\beta}||_2$ are respectively the sum of the absolute value and the sum of square of each element in $\boldsymbol{\beta}$, and λ and α are two tuning parameters. The elastic net is reduced to LASSO when α is 1, and to ridge regression when α is 0.

The elastic net is designed to overcome the limitations of LASSO in two aspects:

- in the "large p, small n" case, which means the number of features are much more than the number of samples, LASSO will select at most n variables before it saturates [1].
- if there are high correlations between a group of features, LASSO will select only one variable from the group [2].

Compared to LASSO, the additional L_2 penalty contributes to the selection of correlated features. Meanwhile, the elastic net also takes advantage of convex optimization, which is well studied and can be solved fast. In this study, the logistic regression with elastic net regularization is implemented by the glmnet [4] package in R.

S2 Results of the elastic net model

We trained the elastic net model using datasets with three sizes: 1-fold (3 million bases), 5-folds and 50-folds. It took about 10 minutes, 30 minutes and 4 hours training the model for the 3 datasets, respectively.

To tune the hyperparameters λ and α, we used the cross-validation method. The λ was tuned by the cv.glmnet function in glmnet package, and α was tuned by maximizing the AUC as described in Methods. Based on the 1-fold dataset, λ and α were tuned to be 4×10^{-6} and 0.1, respectively. The coefficients of the trained model were shown in Table S1. In this set of parameters, the elastic net model merely removed two variables (x_{17} and x_{61}).

We noted that the glmnet package reported a convergence warning and selected no feature at all, in the case of α equals 1, or, the LASSO setting. It was different from the results of the liblinear package (see Table 1). We hypothesized that the difference was caused by the optimization techniques adopted by the two packages.

We assessed the elastic net model in two aspects: consistency and empirical discrimination power. First, following Ewing et al. [5] and Li et al. [6], we assessed the consistency of error rates predicted by the elastic net, as shown in Figure S1. Trained on the 1-fold dataset, the bias between the predicted scores and observed scores of the elastic net was less than that of the AIC and BIC method (see Figure2a). Trained on the 5- and 50-folds data, the elastic net model showed similar consistencies. Compared with the 1-fold data, they both had larger biases when predicted scores were less than 20, and had less biases when predicted scores were larger than 25.

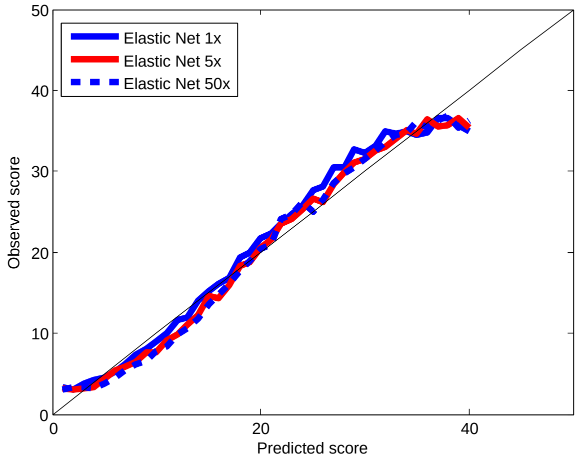

Figure S1: The observed quality scores versus the predicted ones of the elastic net model by different sizes of training sets. The predicted scores, or equivalently, the predicted error rates of the test dataset were calculated according to the model learned from the training dataset, and the observed (aka. empirical) ones were calculated as -10*log10 [(total mismatches)/(total bp in mapped reads)].

Second, we compared the empirical discrimination power of the elastic net with that of the L_1 regularization, as shown in Figure S2. Like the L_1 method,

the empirical discrimination power of the elastic net increased as the sizes of training sets increased. The 5- and 50-folds data respectively increased the empirical discrimination power by 5% and 9% over the 1-fold data on average. Meanwhile the empirical discrimination power of the 50-folds data using the elastic net was close to that of the 5-folds data using the L_1 regularization.

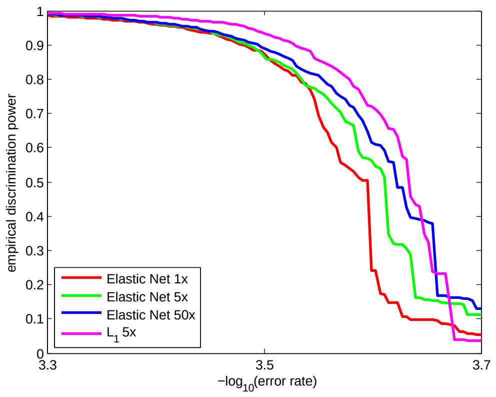

Figure S2: Empirical discrimination powers for the elastic net model with three training sets and for the L_1 regularization with the 5-folds dataset. The x-axis is the -log10(error rate) in the range between 3.3 and 3.7. The y-axis is the empirical discrimination power defined as the largest proportion of bases whose empirical error rate is less than 10^{-x}. 1x, 5x, 50x indicates that the model is trained with 1-, 5-, 50-folds of 3 million bases, respectively.

In sum, the elastic net model indeed selected more features than LASSO did. In fact, almost all features were selected in this dataset. In terms of computational time, consistency and empirical discrimination power, the glmnet package performed no better than the liblinear package did.

x	description	Elastic Net	x	description	Elastic Net
x0	intercept	15.62	x38	A(TG)	-0.26
x1	largest intensity	0.55	x39	C(AC)	2.38
x2	second largest intensity	-3.81	x40	C(AG)	-0.32
x3	average of x1	-0.87	x41	C(AT)	-0.37
x4	average of (x1-x2)	-5.32	x42	C(CA)	-1.23
x5	standard error of (x1-x2)	-1.33	x43	C(CG)	-1.09
x6	1/x3	-2.92	x44	C(CT)	-1.56
x7	x5	0.9	x45	C(GA)	1.03
x8	log(x5)	0.54	x46	C(GC)	0.61
x9		-7.06	x47	C(GT)	0.35
x10		-2.85	x48	C(TA)	0.6
x11		-1.79	x49	C(TC)	-0.16
x12		-0.44	x50	C(TG)	-0.81
x13	piecewise function of $\lvert x_1 - x_2 \rvert$	1.11	x51	G(AC)	0.46
x14		2.69	x52	G(AG)	-0.09
x15		7.85	x53	G(AT)	-0.87
x16		3.72	x54	G(CA)	0.04
x17		-	x55	G(CG)	-0.41
x18	current cycle number	-0.02	x56	G(CT)	-1.34
x19	inverse distance	-0.18	x57	G(GA)	1.73
x20		-1.42	x58	G(GC)	0.18
x21		-1.03	x59	G(GT)	-1.09
x22		-0.21	x60	G(TA)	0.59
x23	indicators of the first 7th cycles	-0.77	x61	G(TC)	-
x24		-0.86	x62	G(TG)	0.42
x25		-1.16	x63	T(AC)	-0.37
x26		2.07	x64	T(AG)	-0.64
x27	A(AC)	-0.34	x65	T(AT)	2.15
x28	A(AG)	-1.18	x66	T(CA)	0.15
x29	A(AT)	-0.99	x67	T(CG)	-1.08
x30	A(CA)	1.35	x68	T(CT)	-0.25
x31	A(CG)	1.1	x69	T(GA)	-0.03
x32	A(CT)	0.24	x70	T(GC)	0.77
x33	A(GA)	1.36	x71	T(GT)	2.03
x34	A(GC)	0.14	x72	T(TA)	-0.31
x35	A(GT)	2	x73	T(TC)	-0.6
x36	A(TA)	0.79	x74	T(TG)	-1.23
x37	A(TC)	0.38			

Table S1: The coefficients of the 74 predictive variables of the elastic net model. We denote these 74 variables by $x = (x_0, x_1, \cdots, x_{74})$. The details of the variables in each row are described in Methods. Meanwhile, '-' implies that the method has removed the feature.

References

[1] Zou, H., Hastie, T.: Regularization and variable selection via the elastic net. Journal of the Royal Statistical Society. Series B: Statistical Methodology **67**(2), 301–320 (2005)

[2] Tibshirani, R.J.: Regression shrinkage and selection via the lasso. Journal of the Royal Statistical Society **58**, 267–288 (1996)

[3] Hoerl, A.E., Kennard, R.W.: Ridge Regression. In: Encyclopedia of Statistical Sciences. John Wiley & Sons, Inc., Hoboken, NJ, USA (1988). doi:10.1002/0471667196.ess2280.pub2

[4] Friedman, J., Hastie, T., Tibshirani, R.: Regularization paths for generalized linear models via coordinate descent. Journal of Statistical Software **33**(1), 1–22 (2010)

[5] Ewing, B., Green, P.: Base-calling of automated sequencer traces using phred. ii. error probabilities. Genome Research **8**(3), 186–194 (1998)

[6] Li, M., Nordborg, M., Li, L.M.: Adjust quality scores from alignment and improve sequencing accuracy. Nucleic Acids Research **32**(17), 5183–5191 (2004)
